# Preprocessing Arabic text on social media

**DOI:** 10.1016/j.heliyon.2021.e06191

**Published:** 2021-02-13

**Authors:** Mohamed Osman Hegazi, Yasser Al-Dossari, Abdullah Al-Yahy, Abdulaziz Al-Sumari, Anwer Hilal

**Affiliations:** a Department of Computer Science, College of Computer Engineering and Sciences, Prince Sattam Bin Abdulaziz University, Al-Kharj 11942, Saudi Arabia; b Department of Computer and Self Development, Preparatory Year Deanship, Prince Sattam Bin Abdulaziz University, Al-Kharj 11942, Saudi Arabia

**Keywords:** Natural language processing, Information extraction, Information retrieval, Database, Data analysis, Knowledge discovery, Sentiment analysis, Document and text processing, Arabic text

## Abstract

**C**urrently, social media plays an important role in daily life and routine. Millions of people use social media for different purposes. Large amounts of data flow through online networks every second, and these data contain valuable information that can be extracted if the data are properly processed and analyzed. However, most of the processing results are affected by preprocessing difficulties. This paper presents an approach to extract information from social media Arabic text. It provides an integrated solution for the challenges in preprocessing Arabic text on social media in four stages: data collection, cleaning, enrichment, and availability. The preprocessed Arabic text is stored in structured database tables to provide a useful corpus to which, information extraction and data analysis algorithms can be applied. The experiment in this study reveals that the implementation of the proposed approach yields a useful and full-featured dataset and valuable information. The resultant dataset presented the Arabic text in three structured levels with more than 20 features. Additionally, the experiment provides valuable information and processed results such as topic classification and sentiment analysis.

## 1 Introduction

Social media has evolved to become an important driver for acquiring information in different domains. Development and application or computerized tools to extract knowledge and information from Arabic text on social media platforms is a complex task. To this end, several methods including text cleaning, natural language processing (NLP), normalization, learning algorithms, and application design tools must be developed [1, 2, 3].

### 1.1 Arabic language

A summary of the several factors that make research work in Arabic NLP (ANLP) interesting and challenging is presented below.

#### 1.1.1 The culture and history of the Arabic language

The history of the Arabic language is unique because it has remained unchanged for more than sixteen centuries (before The Holy Quran in 609 CE). Arabic is a Semitic language with an inseparable link with Islam and the Arabic culture, where it is the language of the holy book for all Muslims (more than 1.62 billion people). Moreover, it is the native language of more than 422 million speakers [3, 4, 5].

#### 1.1.2 Characteristics of the Arabic language

In addition to the uniqueness of the historical and cultural background of the Arabic language, its nature and structure are different from other languages such as English. For example, this language is written from right to left. It comprises 28 letters, including three vowels and diacritics (short vowel symbols inscribed atop regular letters). Diacritics may affect the semantics and syntax of a word [6], as shown in Table 1.

**Table 1:** Arabic diacritics.

Diacritic Name	Diacritic Symbol	Pronunciation
fatha		Short "a"
damma		"uu"
kasra		Short "i"
tanween fatha		"an"
tanween damma		"un"
tanween kasra		"in"
hamza		
hamza above Alif		"a'a"
hamza under Alif		"i' i"
shada		It comes with the diacritic to increase its sound

The shapes of Arabic letters change according to their positions in a word, and there is no capitalization in Arabic. Arabic is a structured language with an abundance of vocabulary, wherein morphology plays an important role. Furthermore, here, words are often constructed in a complex manner. Moreover, it may contain affixes, agglutinative, and drop features [3, 4, 5, 7].

#### 1.1.3 Types of Arabic languages

There are three variants of the currently used Arabic language [7, 8]:

1. Classical Arabic (CA) is the oldest type of Arabic. It is the language of The Holy Quran, Islamic books and media, and Arabic literature. This variant is commonly taught in Arabic schools and universities. CA is restricted to religious and highly formal contexts.
2. Modern standard Arabic (MSA) is a direct descendant of CA and is now the language used in elevated discourse and correspondence, contemporary literature, and mass media (newspaper, radio, television, and Internet). MSA is largely uniform throughout the Arab world, and it being a simplified version of CA, can be comprehended by several people.
3. Dialect Arabic language, which refers to the regional dialects, is used in daily-life communication including informal exchanges, discourse, and popular culture media. It is the language used by speakers on social media, discussion forums, and short messaging. Arab people from one region can generally understand the dialects from other regions. Broadly speaking, there are four major dialect groups in the Arab world today: (1) Egyptian (Egypt), (2) Maghrib (Morocco, Mauritania, Algeria, Tunisia, and Libya), (3) Gulf Area (Saudi Arabia, UAE, Kuwait, Oman, Bahrain, Qatar, and Yemen), and (4) Levant (Lebanon, Syria, Jordan, and Palestine).

Regarding the processing of Arabic text, these varieties of currently used Arabic languages can be categorized into two groups: (1) standard Arabic text (CA or MSA) and (2) non-standard Arabic text, which may be dialect or mixed, such as social media. This is owing to the fact that the algorithm and tools used in processing CA text can be applied for processing MSA because both languages are well-structured and written in standard Arabic language format. Therefore, in this study, we use these two varieties (standard or non-standard).

#### 1.1.4 Challenges in processing Arabic text

The complexities and characteristics of the Arabic language have made processing Arabic text, a major challenge, wherein researchers must deal with several difficulties, such as ambiguity, diglossia, and the difficulties in reading and understanding the Arabic script: (i) the Arabic letter changes according to its position in the word, (ii) no capitalization or dedicated letter, and (iii) the complex structure of the word and morphology. Another challenge is the normalization of inconsistency in the use of certain letters, dialect words, or diacritical marks.

Tackling the difficulties in NLP tools such as tokenization, stemming, and morphology analysis is yet another challenge faced by researchers and developers.

These issues have been widely studied [5, 7, 9, 10].

### 1.2 Social media Arabic Text

Social media Arabic text has special characteristics compared to those of CA, MSA, and dialect Arabic. Such text can be a mixture of all these varieties, and additionally, it may contain non-Arabic words, samples, notations, and orthographic features such as spelling mistakes, repeated letters, or express emotive words. Therefore, in addition to tackling the complexity challenges of the Arabic language, other issues such as cleaning noise from the text, changing a word to its normal form, dealing with the dialect language must be handled. Consequently, preprocessing social media Arabic text poses a great challenge to the research and application developers.

### 1.3 Description of problem

This study aims to provide solutions to the problems in preprocessing social media Arabic text and aims to make information available to Arabic speakers in their native languages (see [5] and [10]).

It is based on the following assumptions.

1. Success in preprocessing social media Arabic text means transferring non-standard Arabic to standard Arabic (CA or MSA), which leads to successful results while extracting information or analyzing data.
2. Processing the cleaned text by applying NLP tools such as tokenization, stemming, and morphology analysis will enhance the text (make it more understandable) and make it available in a computational format because it fits the nature of the Arabic language (Arabic language is a structured language with an abundance of vocabulary, where morphology plays an important role).
3. Providing an approach for integrating all these solutions into a single framework will be a standard solution for all the challenges in handling social media Arabic text (preprocessing and NLP difficulties). Going forward, this will be a base for models and application development.
4. Providing the final dataset in the form of relational database tables will make the information available to a variety of developers and users.

### 1.4 Contribution

Although research interest in preprocessing Arabic text on social media has increased recently, most of this research focuses on preprocessing Arabic text for special purposes such as sentiment analysis or dealing with dialect language. Standard or integrated work on preprocessing Arabic text on social media that aims to make data available for processing and information extraction is still lacking. Particularly, the tools and frameworks that can produce full-featured datasets are required.

This paper proposes an easily implementable and standard approach for preprocessing Arabic text on social media and producing full-featured datasets that can be suitable for all types of knowledge discovery algorithms. This approach endeavors to convert social media data from raw or source-based data to computation-based data.

To the best of our knowledge, this is the first study that aims to provide a framework (an integrated approach) for dealing with social media Arabic text and merge the concepts of data manipulation and database.

Most of the previous studies process the Arabic language based on the English language processing methodologies without considering the structured nature of the Arabic language, mainly, the abundance of vocabulary in it. This study provides a morphology algorithm that generates word vocabulary based on Arabic morphology templates.

## 2 Related works

Although handling Arabic text has recently received increased attention from researchers, most of their works that provide solutions to the problems in processing Arabic text are concerned with MSA, such as [11, 12, 13, 14]. Majority of the recent research works on social media Arabic text that provide solutions to the problems encountered in social media Arabic text processing focus on preparing data to specific objectives, mainly sentiment analysis. However, some of these studies presented satisfactory solutions in the area of social media Arabic text processing, e.g., Darwish et al. [3] improved the retrieval of Arabic text from microblogs by enhancing the normalization of Arabic microblogs on Twitter. They first considered the normalization of Arabic letters, and then expanded their normalization processes to handle words using processes such as normalizing elongated and shortened words, removing stop words, stemming, and considering Arabic dialects. Al-Twairesh et al. [15] collected the dataset for their study from Arabic tweets and then applied existing methods to clean and preprocess the collected dataset. They removed the URLs and user mentions (@user) and normalized the Arabic letters (أ, ة, ي,و). Then, they annotated the data for sentiment analysis. Refaee and Rieser [16] developed an Arabic Twitter corpus for subjectivity and sentiment analysis. They used the Twitter application program interface (API) to collect tweets in real time and cleaned the extracted data by eliminating the Latin characters. Alshutayri and Atwell [17] worked on developing a corpora of dialect text to be used in ANLP. They created a corpus of dialect Arabic text from Twitter using common dialect words (seeds). They used the Twitter API to connect to Twitter and collected the tweets using the seed words. Similarly, Mubarak and Darwish [18] worked on obtaining a multi-dialect Arabic corpus from Twitter. They used the Twitter API to obtain Arabic tweets and then extracted user locations to classify the text into specific dialects according to the extracted locations. Furthermore, they preprocessed the Arabic characters for converting them into normal forms. Alkhatib et al. [19] provided a framework for incident and event monitoring in smart cities. Their framework is based on Arabic social media text, and the data were preprocessed by removing the stop words prepositions (من,إلى and عن), conjunctions (و ، أو), and user accounts. Stemming is used to tackle unclassified words. Al-Ghadir and Azmi [20] presented a study of Arabic social media, in which, considerable effort in preprocessing the data is seen. The authors removed the characters not belonging to the Arabic language (including digits and marks), unified the different forms of the Arabic character such as (أ، إ ، ا), and then normalized the repeated characters. They used a tokenization and stemming algorithm to provide a stem version of the topic. Kaity and Vimala [21] proposed a method for building a sentiment lexicon using unannotated corpus. The rationale behind their method is to use an English seed sentiment lexicon for developing Arabic sentiment lexicon. During preprocessing, they clean the data by removing the comments containing “links” or “symbols” and remove stop words and prepositions. They use lemmatization to convert the words into their roots or dictionary forms.

Sentiment analysis has gained the highest attention from researchers working on social media Arabic text; however, most of their works focus on employing machine-learning and deep learning techniques to provide solutions to the problems in sentiment analysis, without concentrating much on the problems of social media Arabic text. Some of them used a readymade dataset and others provided prepressing approaches using common tools, e.g., Hammad & Al-awadi [22] employed a support vector machine (SVM), back propagation neural networks (BPNN), naïve Bayes (NB), and decision tree (DT) for hotel reviews on social media. The authors created their dataset from Facebook, Twitter, and YouTube comments. They preprocessed the data by removing stop words and punctuations and employed a light stemmer to filter some features. Al-Rubaiee et al. [23] used NB and SVMs to study the opinions of Saudi tweeters regarding Mubasher products. They cleaned the data by removing stop words and filtered them using tokenization and stemming algorithms. In [24], NB, SVM, and k-nearest neighbor (KNN) were applied to analyze the sentiment of Twitter Arabic text. Here, the authors preprocessed the data by removing stop words and processed them using a tokenization and stemming algorithm. In [25], SVM and NB were used in several scenarios to conduct and analyze the sentiment analysis for the Jordanian Twitter Arabic text corpus. The authors used the ASAP utilities (Excel add-in utilities) to preprocess the data. They removed links, duplicate tweets, hashtags, and foreign characters. They used the RapidMiner software for NLP (tokenization, filtration of tokens, and Arabic stemming). In [26], SVM and NB were used in the sentiment analysis of dialectical texts from Facebook comments. The authors proposed an approach to preprocess the text based on each language category (MSA or dialect). They applied a stemming algorithm for dialectical texts to consider the heterogeneity between the dialectical texts and the MSA text.

Recently, deep learning techniques have been used in sentiment analysis research. Some of these studies use preprocessing or NLP in preparing their data. For example, in [28], the text was tokenized and then translated into English before applying the deep learning algorithms. In [29], all diacritics Arabic text was removed. The stop words, punctuations, numbers, symbols, and non-Arabic words were replaced by keywords. Only three Arabic characters (أ, ة, ى) were normalized.

Table 2 presents a summary of recent research works on social media Arabic text in sentiment analysis.

**Table 2:** Research studies on sentiment analysis in social media Arabic text.

Study	Year	Approach	Data	Preprocessing	Processing (NLP)
[22]	2016	Machine learning (SVM, NB, DT)	Hotel reviews on social media	Removing stop words and punctuations	Stemming
[23]	2016	Machine learning (SVM, NB)	Twitter	Removing stop words	Tokenization and stemming
[24]	2016	Machine learning (SVM, NB, KNN)	Twitter	Removing stop words	Tokenization and stemming
[25]	2017	Machine learning (SVM, NB)	Twitter	Removing links; duplicating tweets, hashtags, and foreign characters	Tokenization and stemming
[26]	2018	Machine learning (SVM, NB)	Facebook comments	Cleaning noise and normalizing	Tokenization and stemming
[27]	2018	Deep learning (convolutional neural network (CNN))	Books; tweets; restaurant, hotel, and movie reviews	No preprocessing (Converting text to vectors and encoded characters (character level features))	No NLP processing
[28]	2018	Deep Learning (CNN-LSTM)	Twitter	No preprocessing (using public data)	Tokenization and translating Arabic to English
[29]	2019	Deep learning (combining differential evolution (DE) algorithm with CNN)	Twitter and other public data	Cleaning and normalizing the Arabic text.	No NLP processing

## 3 Proposed approach

**Figure 1:**
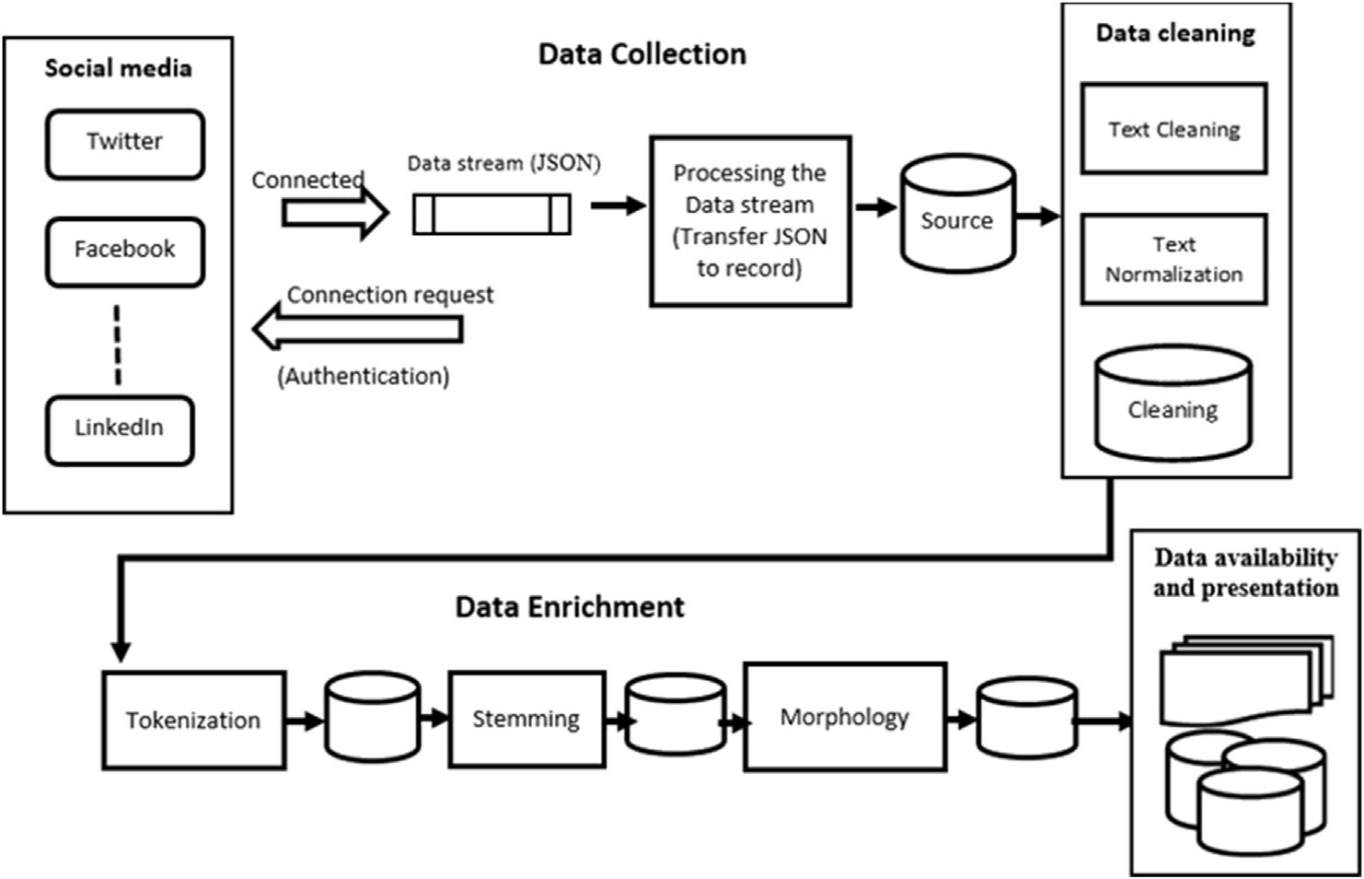
The framework of the proposed approach.

The proposed approach is based on the following four stages (Figure 1).

1. Collecting data.
2. Cleaning and normalizing the Arabic text (prepressing).
3. Enriching the Arabic text (NLP).
4. Presenting the final data.

### 3.1 Data collection

Social media involves a variety of structures, components, web links, and data types such as videos, audios, and text. Our approach focuses on collecting only Arabic text without considering the nature of social media platforms or other components.

**Figure 2:**
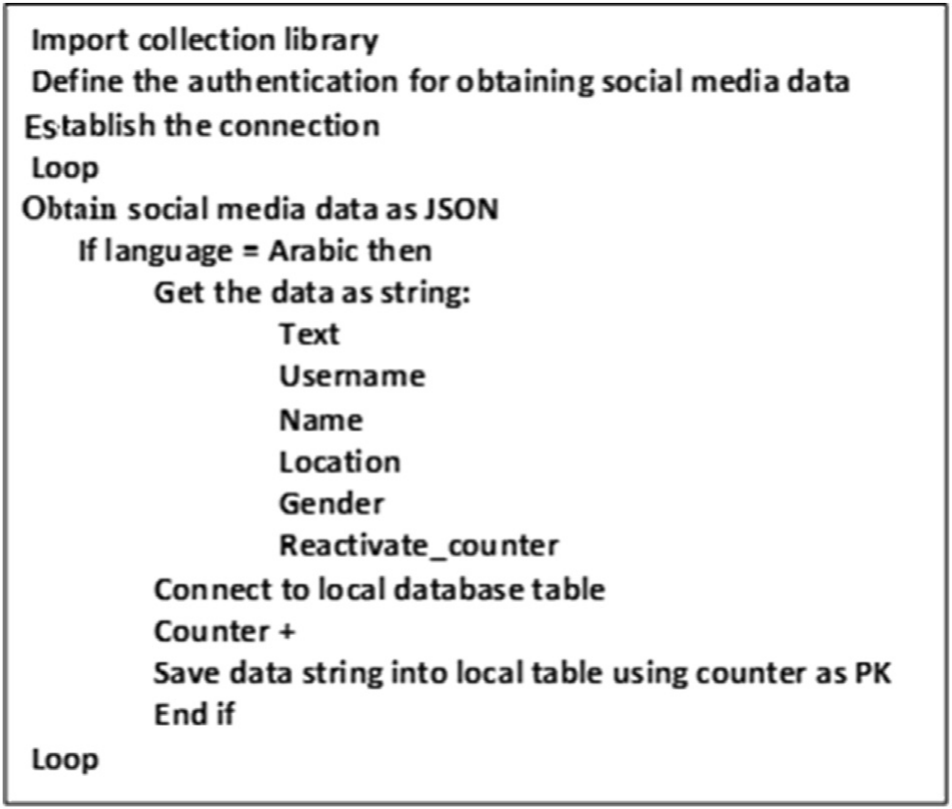
Data collection algorithm.

At this stage, Arabic text data are collected from social media servers and stored in a local database file. This approach provides reliable and valid text data. It involves three phases: data discovery, connection, and storage, as shown in Figures 1 and 2.

#### 3.1.1 Schema identification (data discovery).

In social media, microblogs are the main data wherein all the text or other data are presented. Most of the existing approaches dissect the content of the microblogs and ignore the user and other information. Our approach focuses on other information such as user, location, data, and time information.

#### 3.1.2 Connection

Several tools such as log files, affiliation with the social media company, or APIs can be used to connect to social media servers. APIs are a back-end interfaces through which developers can connect to existing services. They are commonly used and publicly available. Most social media platforms make their data available through their APIs; therefore, in this approach, we use social media API authentication tools to connect to social media services. For example, in our experiment, we used Twitter OAuth, “An open protocol to allow secure authorization in a simple and standard method from web, mobile and desktop applications” [30] to connect to the Twitter server. Similarly, we can use the Facebook GraphAPI to connect to the Facebook server.

#### 3.1.3 Data collection and storage

Most APIs over social media data are in JSON (JavaScript Object Notation) format, which makes them easy to analyze. Therefore, in this phase, data are collected online as JSON through social media API. The data collection algorithm (Figure 2) sends a query to the social media database using social media API (e.g., Tweepy API wrapper in Twitter). The API returns the query as a JSON object. The algorithm then decodes the JSON object and converts it into a string.

### 3.2 Data cleaning

According to Oueslati et al. [10], Arabic text preprocessing is an essential step in any ANLP application. Preprocessing Arabic text on social media, which is usually informal (not standard), is more complicated owing to several reasons such as the presence of dialect text, common spelling mistakes, extra characters, diacritical marks, and elongations. Consequently, to preprocess such Arabic text, we must perform additional processing such as stripping the elongations, diacritical marks, and extra characters. Additionally, we must convert the text into its normal Arabic form. To this end, this approach provides two preprocessing modules:

a Text cleaning
b Text normalization

#### 3.2.1 Text cleaning

Most Arabic text on social media contains noise such as elongations, diacritical marks, extra symbols, and/or mixed language. The first and the most important step in preprocessing is cleaning Arabic text by removing such noises.

Most of the previous algorithms clean the noises by expecting all possible noises and then searching each noise in the text and cleaning it, which makes the cleanliness of the text depend on the degree of anticipation of noises. This method of cleaning may not provide accurate results because it is not easy to expect all noises, in addition to the possibility of introduction of new noises. Our cleaning algorithm works differently than the previous algorithms—it does not look at the noises but only selects the required text.

**Figure 3:**
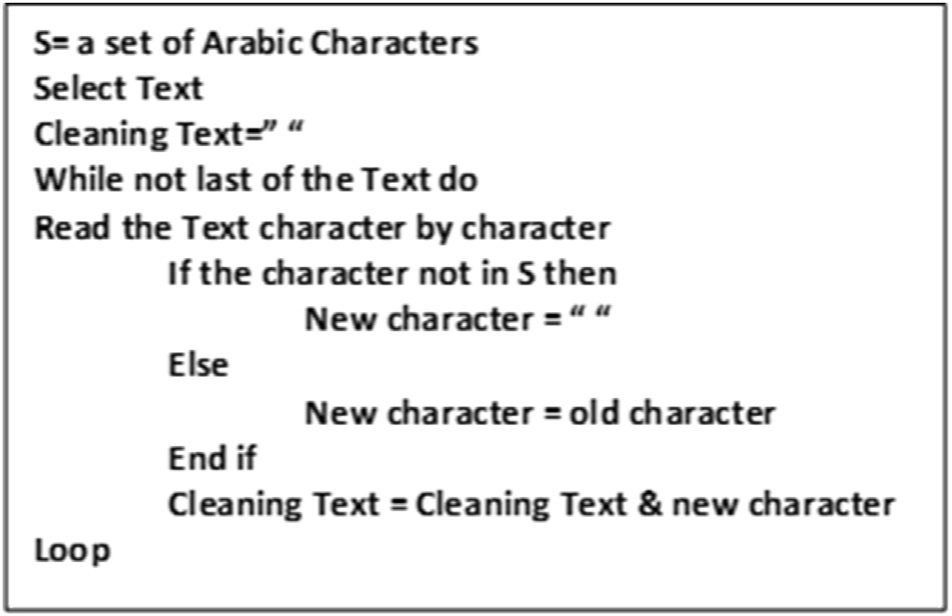
Cleaning algorithm.

As shown in Figure 3, the cleaning algorithm reads the text, character by character, and then checks whether the character belongs to the Arabic alphabet or the required character; if yes, the algorithm selects it, otherwise, replaces it with a space.

This method of cleaning eliminates all noises, such as additional characters, samples, non-Arabic characters, URLs, and any shapes, and provides clean Arabic text without affecting its meaning or content. Moreover, it transforms every letter into its standard form, e.g., “alif” has several forms “أ”, “إ”, “آ”, and “ا”. Given this, the module provides the possibility of choosing additional characters if required.

According to Jian-qiang and Xiao-lin [31], removing stop words, numbers, and URLs is appropriate for reducing noise and does not affect the performance of sentiment analysis, which we think is more sensitive to these components than the remaining processing algorithms such as information extracting and mining algorithms.

#### 3.2.2 Text normalization module

After cleaning the tweet text, the second step is to change the text into its normal form. Some Arabic words on social media are written in non-standard ways, e.g., some words contain repeated characters such as “مبرووووووووك ” instead of “مبروك”, which means congratulations, emotions such as “ههههههه” which indicate laughing, and include common spelling mistakes and/or dialects.

Most of the previous works [3, 18, 19] did not provide individual algorithms for normalization; they used a single algorithm for both cleaning and normalization. In our approach, we considered normalization to be a different preprocessing task because we used a different approach while cleaning the noises. Our proposed normalization algorithm replaces a non-normal word by a normal one by removing the repeated characters and using a set of common non-normal words.

### 3.3 Data enrichment (enhancing Arabic text)

Although applying NLP tools for Arabic text enriches the text, enhancing the meaning and providing more information from this text by giving meaning to the text word and by adding synonymous words can further enrich this text. However, this issue did not receive much focus in previous studies because (i) it is difficult to develop NLP for this type of Arabic text and (ii) most of the previous works followed the English language processing approaches, where morphology is not significant unlike that in Arabic language.

This section discusses the application of NLP tools in social media Arabic text, assuming that the implementation of these tools for Arabic social media text will enrich the text, enhance its meaning, and provide a useful and full-featured dataset. Additionally, it serves our objective of providing a complete solution through a single framework or approach (integration solution).

Three related modules apply the NLP tools for cleaning Arabic text (Figure 1).

#### 3.3.1 Tokenization

According to Faragly and Shalan [5], tokenization in Arabic is problematic owing to the complexity of the structure of the Arabic language, e.g., an Arabic name has no capitalization and may have a middle token such as “ بن ”; some Arabic words may have up to three different tokens.

Based on the cleaning and normalization results provided by the above algorithms, the tokenization algorithm can be implemented easily and in a straightforward manner.

**Figure 4:**
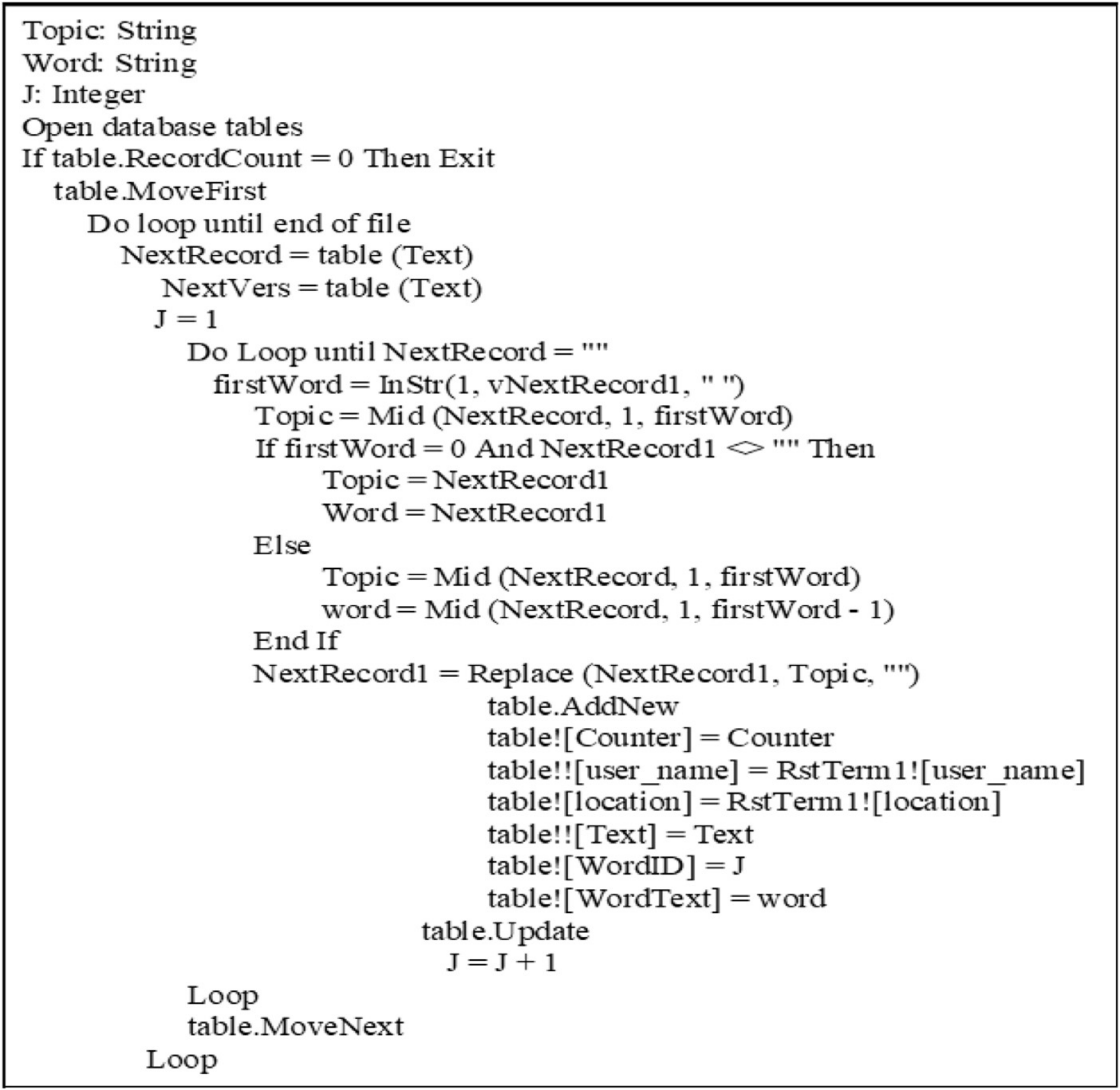
Tokenization algorithm.

The tokenization algorithm uses the spaces between words and punctuation, such as stop marks, commas, and semicolons to fragment text into words. Then, it stores each word in an individual database field (Figure 4).

#### 3.3.2 Stemming and root generation

There are several Arabic stemming algorithms. According to Sawalha et al. [32], each stemmer is based on different text corpora, and most of them are designed to work for MSA; therefore, the application of any one of them in our approach may be difficult, especially, because our approach aims to provide an integrated solution.

This subsection proposes a special stemmer/root generating algorithm to handle the text words that result from the above stage (the tokenization stage). Certain words that result from applying the previous algorithm may contain dedicated words or concatenated letters or may be written in an informal manner, e.g., in some words, “و” which means and, is connected to the word without using the space character. Therefore, an Arabic stemmer module that can generate word stems, word roots, and special word identifiers is proposed.

**Figure 5:**
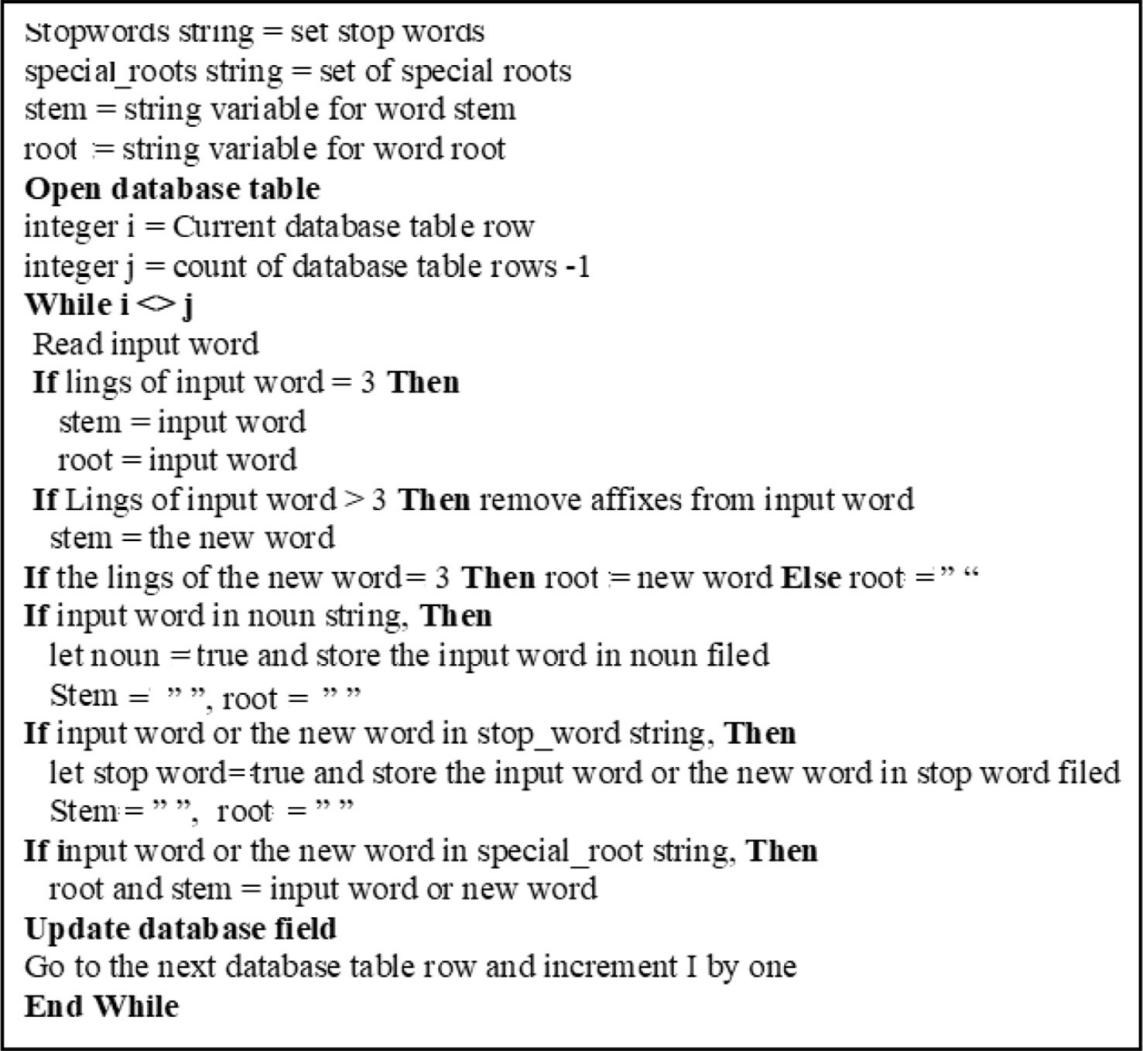
Stemming and Root generation algorithm.

The proposed stemmer module (Figure 5), provides a solution to the challenges resulting from the complexity of social media Arabic words and aims to fulfill two objectives: (1) help understand the meaning of the word by providing its root (2) determine whether the word is a noun, stop word (preposition or pronoun), or a non-standard Arabic word (in case of not finding its root), a dialect, an error, or a non-Arabic word, yet written using the Arabic script. The proposed stemmer algorithm functions as follows:

(1) Provides sets of common Arabic names (nouns), prepositions, pronouns, and special roots (roots with more than three characters).
(2) Checks if the word is in one of the abovementioned sets; if yes, the algorithm retains the word as is and specifies it in the appropriate data field.
(3) If the word is not present in one of these sets, the algorithm checks to determine whether the word has three characters; if yes, the root and the stem are the same and are equal to the word. If the word has more than three characters, the algorithm checks if there is/are extra character/s that is/are concatenated with the word, such as ‘و’. If yes, it removes it/them and returns to step (2), otherwise goes to step (4).
(4) Removes the affixes from the word (the vowel letters at prefixes, suffixes, and infixes), and then determines if the result provided known root (three-character word) or did not provide known root, in which case, the word is considered it an unrecognized Arabic word or a dialect word.

#### 3.3.3 Morphological generation

**Figure 6:**
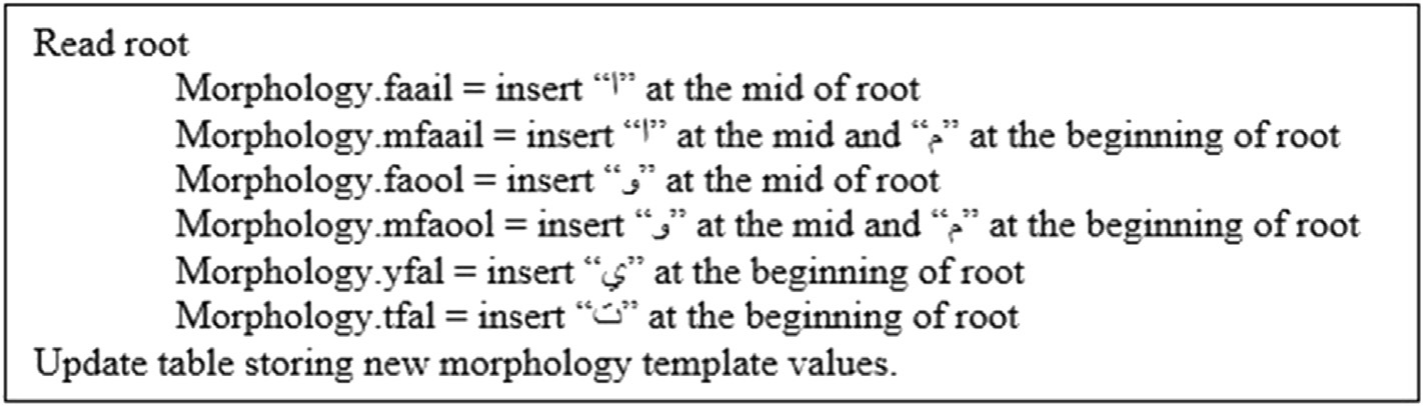
Morphology generation algorithm.

In this approach, the nature of the Arabic language is considered by generating word vocabulary. In this module, six word vocabularies are generated using Arabic morphology templates, “فعل“, “فاعل“, “فعول“, “مفعول“, “مفاعيل“ and ”يفعل”. The morphology generation algorithm inserts or adds letters to root words at certain positions according to a morphology template (Figure 6).

### 3.4 Data availability

According to our approach, preprocessing of the source data will result in the final dataset, wherein all social media Arabic text will be available for processing. The dataset will then be analyzed and used for information extraction. Therefore, the final dataset is designed and structured to occupy a database table with nearly all the features (more than 20 features are included—Figure 15).

## 4 Experiment

Twitter was chosen to implement our approach and for a functional experiment because Twitter is a powerful social media network with millions of people using it as part of their daily lives and routines. In the Arabic world, Twitter, which provides valuable content has been used for political communication, marketing, advertising, business purposes, entertainment, knowledge sharing, news, and sports. Moreover, it offers a well-designed API. Additionally, Twitter provides a platform for developers that includes communities, customized sets of tools, and well-written documentation [33].

### 4.1 Collecting Twitter Arabic text

**Figure 7:**
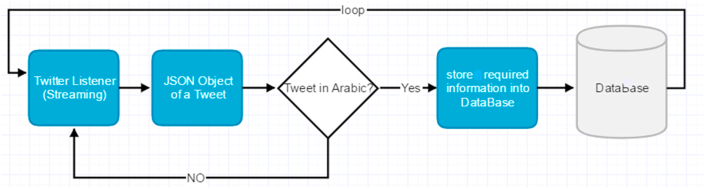
Twitter data collection.

After designing the database table, we used Twitter OAuth to connect to the Twitter server. The connection algorithm sends a query to the Twitter database using Twitter’s API through the Tweepy API wrapper. Twitter returns the query as a JSON object. The algorithm then decodes the JSON object and converts it into a string (Figures 2 and 7).

**Figure 8:**
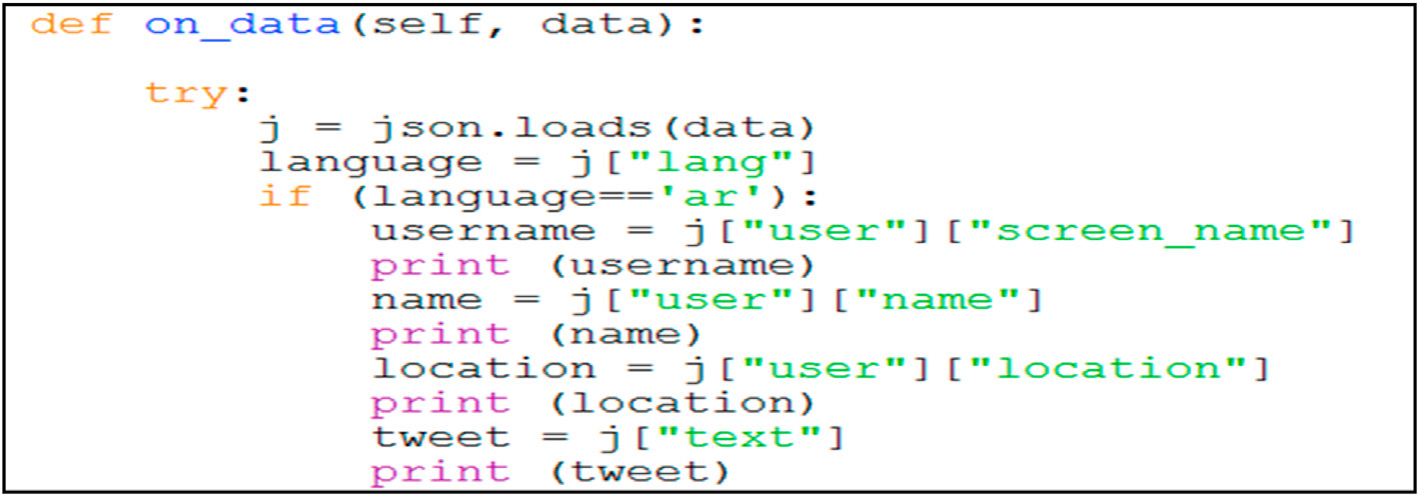
Screen snapshot of data collection code.

As illustrated in Figure 8 and mentioned in section 3.1, the data collection algorithm collects the tweet data through an infinite loop as follows (Figures 7 and 8).

1. Obtain tweet data as JSON objects.
2. Use the Tweepy interface for data streaming.
3. Check whether the text is in the Arabic language.
4. Store the collected data in the database table and proceed to step 1.

### 4.2 Cleaning Twitter Arabic text

**Figure 9:**
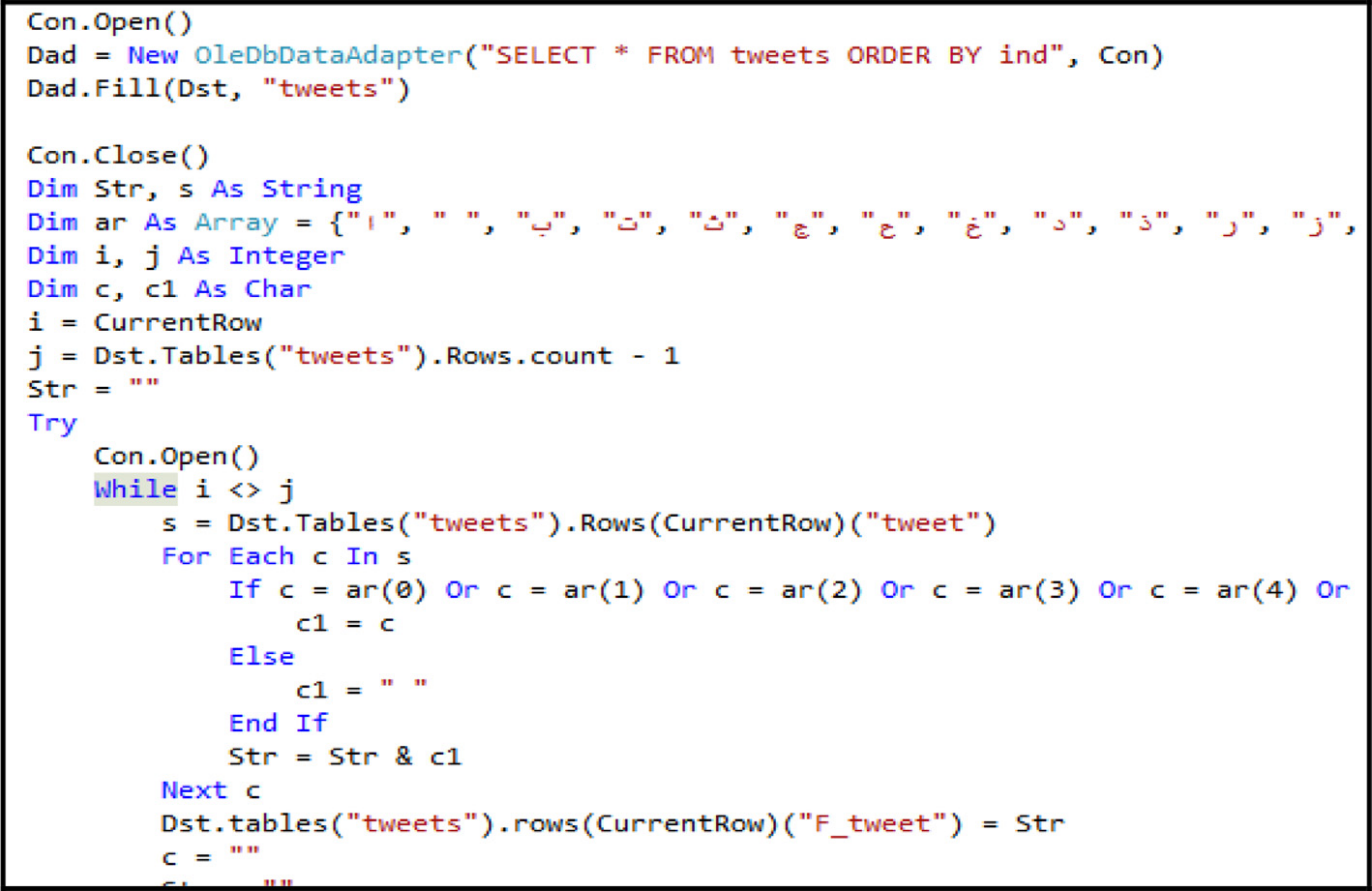
Screen snapshot of text cleaning code.

**Figure 10:**
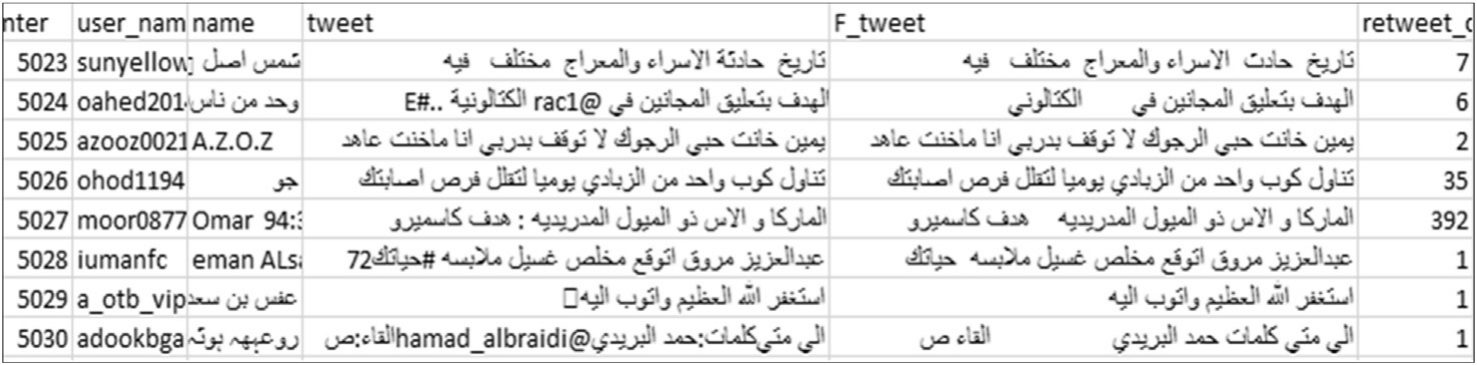
Screen snapshot of sample cleaning data.

The following is an example illustrating the appearance of the text after applying the cleaning algorithm (Figure 3). Figure 9 presents a snapshot of the implementation of the cleaning algorithm on a tweet text. A snapshot of the final cleaning result is illustrated in Figure 10.

Text before cleaning:

الهدف بتعليق المجانين في @rac1 الكتالونية ElClasico #Messi

Text after cleaning:

الهدف بتعليق المجانين في الكتالونية

**Figure 11:**
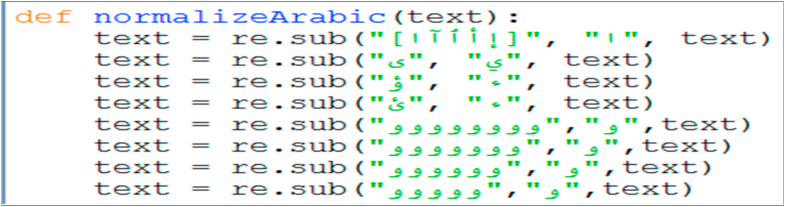
Screen snapshot of normalization code.

After cleaning the tweet text, as the second step, the text is converted into its normal Arabic language form. In this experiment, we consider words that contain repeated characters and words that have some common mistakes and unnecessary spaces (Figure 11). In future work, this set will be expanded and implemented in a database table to act as a dictionary of non-standard and dedicated Arabic words.

### 4.3 Enhancing the collected Twitter Arabic text

Implementation of the enrichment algorithms on the Twitter text is done as follows.

1. Tokenization: Twitter Arabic text: The cleaned tweet text is first fragmented into words and stored in a database table. Figure 12 illustrates sample fragment words after applying the tokenization algorithm in Figure 4.

**Figure 12:**
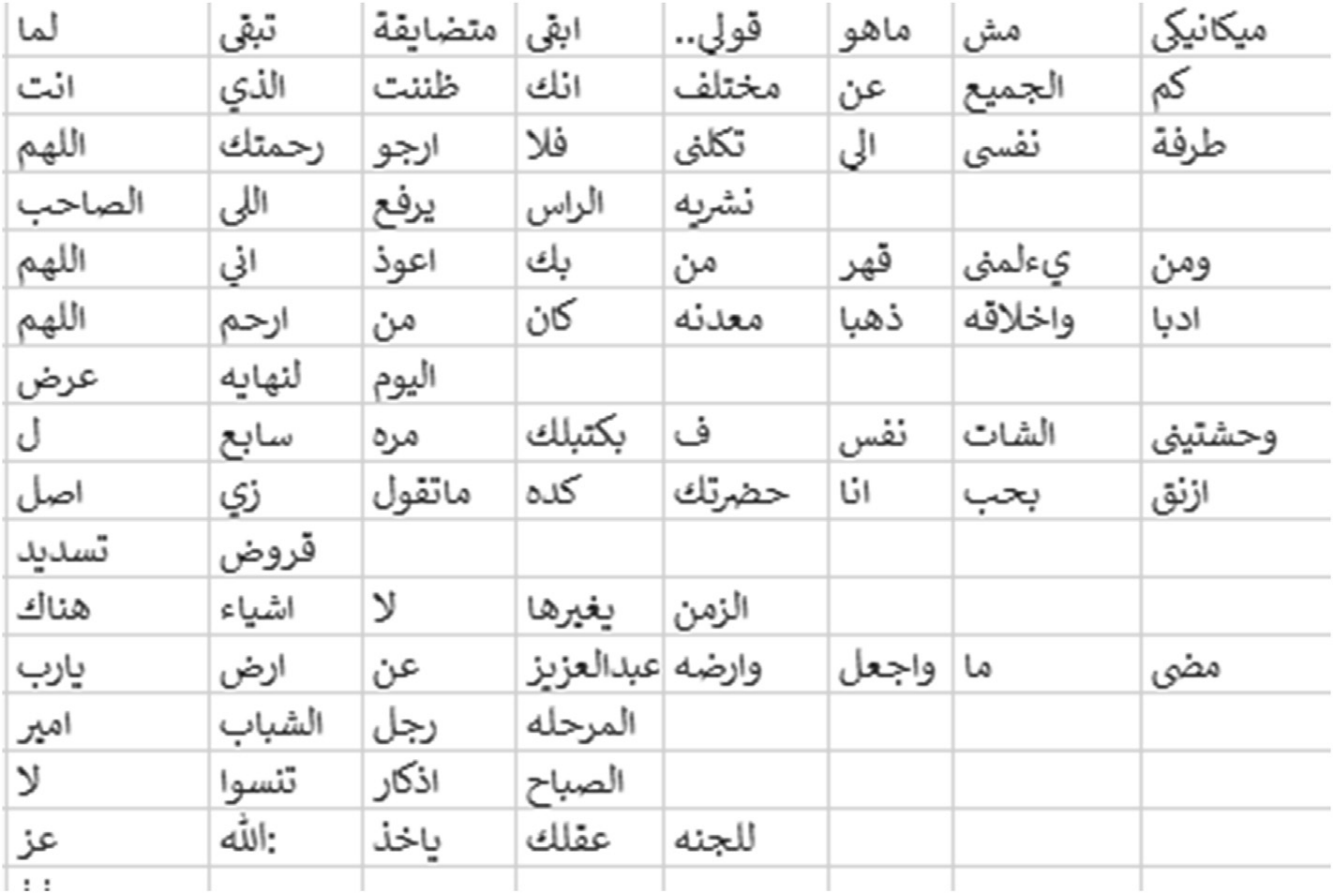
Screen snapshot illustrating a sample of the fragment words after applying tokenization algorithm.
2. Stemming: After fragmenting the tweet text (tokenization), the stemming algorithm (Figure 5) is applied. The results reveal that the algorithm works satisfactorily, where almost 70% of the words provide accepted stemming and roots. The other words are classified as either stop words, nouns, unrecognized Arabic words, or a dialect word. Figure 13 presents the screen snapshot of the stemming code, and Figure 17 presents that of the database showing sample results.

**Figure 13:**
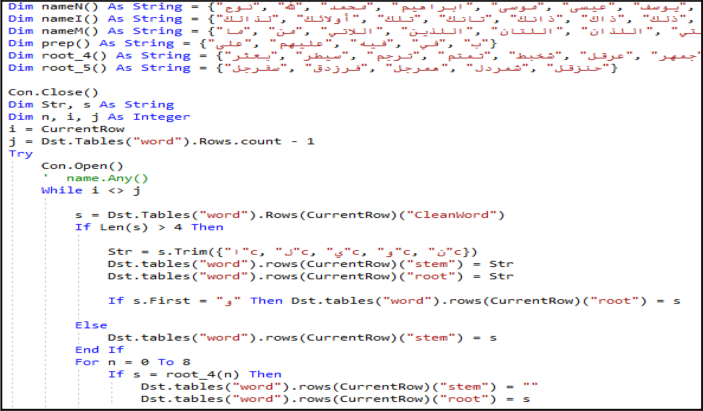
Screen snapshot of the stemming and root generation code.
3. Morphological generation: Finally, we applied the morphology generator algorithm (Figure 6) to generate word morphology templates, “فاعل”,“فعول”, “مفاعيل”, “مفعول” and “يفعل”. Figure 14 presents the screen snapshot of the morphology generator code, and Figure 16 presents a screen snapshot of the database that presents the sample results.

**Figure 14:**
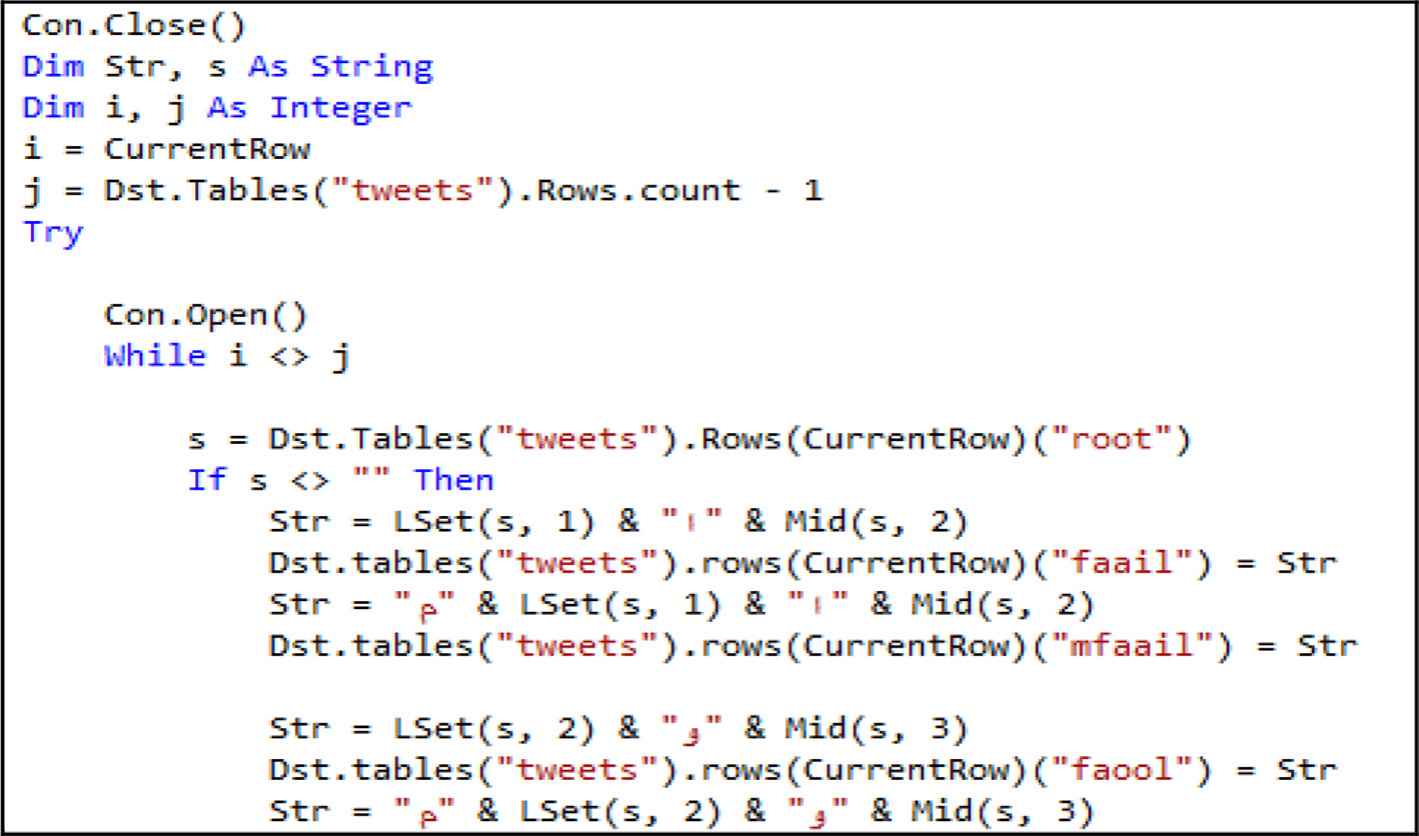
Screen snapshot of morphology generation code.

### 4.4 Final Twitter data

The final dataset in our experiment was generated in the following stages.

**Figure 15:**
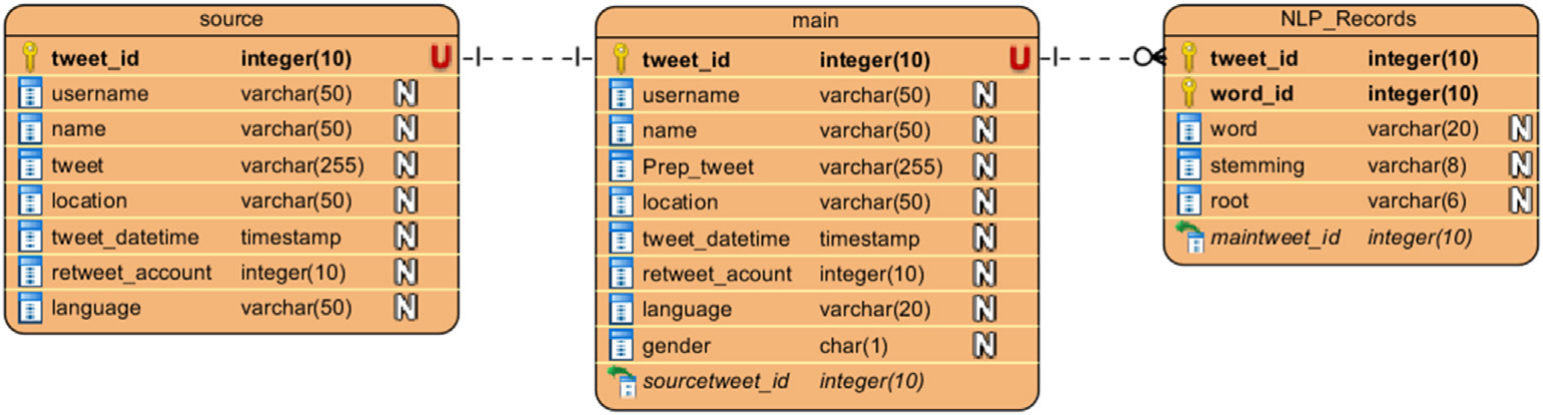
Database schema.

1. After connecting to the Twitter server, the source data are collected as a stream of JSON strings and immediately stored in a database table (Figures 2 and 7). This stage results in the source data and its continuous stage. All data are stored temporally in a single database table.
2. In the second stage, preprocessing algorithms (cleaning and normalization) are applied to the source table, which generates a new database table with cleaned tweet as a new field (Figure 10). This new database table is used as the final storage for Twitter data.
3. In the third stage, the new database table is manipulated using ANPL to provide additional features.
4. In the fourth stage, the final dataset is obtained. It presents the data in many layouts and tables according to the requirements of processing algorithms (Figures 10, 15, and 16). Additionally, the data are made available in several formats such as text, MS Excel, MS Access, SQL Server, and Oracle files.

**Figure 16:**
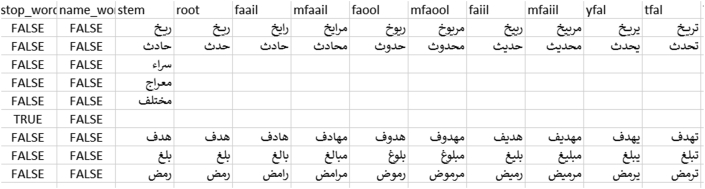
Screen snapshot of database table presenting stemming, root, and morphology processing results.
5. In the final stage, the implementation of any information-extracting algorithm becomes easy. For example, the following can be implemented.
  • Database queries to (i) obtain different types of information such as search results and (ii) select and/or provide reports on specific or general information.
  • Analysis algorithms
  • Visualization techniques to present visual representations of the data, such as plots and graphs.
  • Predictive or descriptive algorithms to extract hidden patterns from these data to obtain more useful results or perform additional studies.

### 4.5 Results and discussion

The experiment was developed and tested using VB.NET, Python, MS Access, SQL Server, and MS Excel. The data presented in this paper were collected online in April 2017. As illustrated in section 4.4, our approach provided a useful and full-featured dataset (Figures 10, 15, and 16), where the Arabic text on Twitter became available as the fields of a database table with other fields, such as name, location, date, and time.

**Figure 17:**
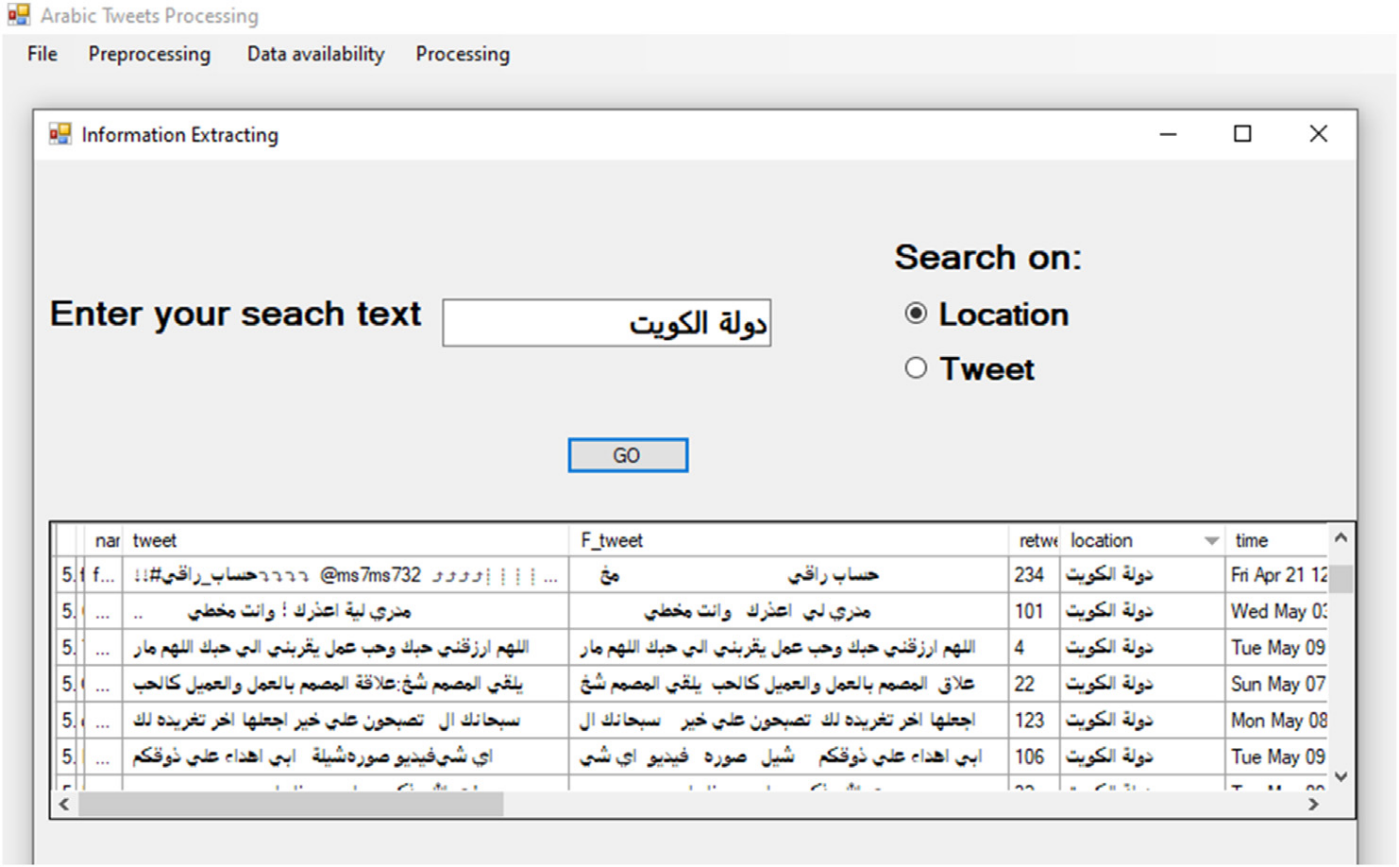
Example of information retrieval application and results.

**Figure 18:**
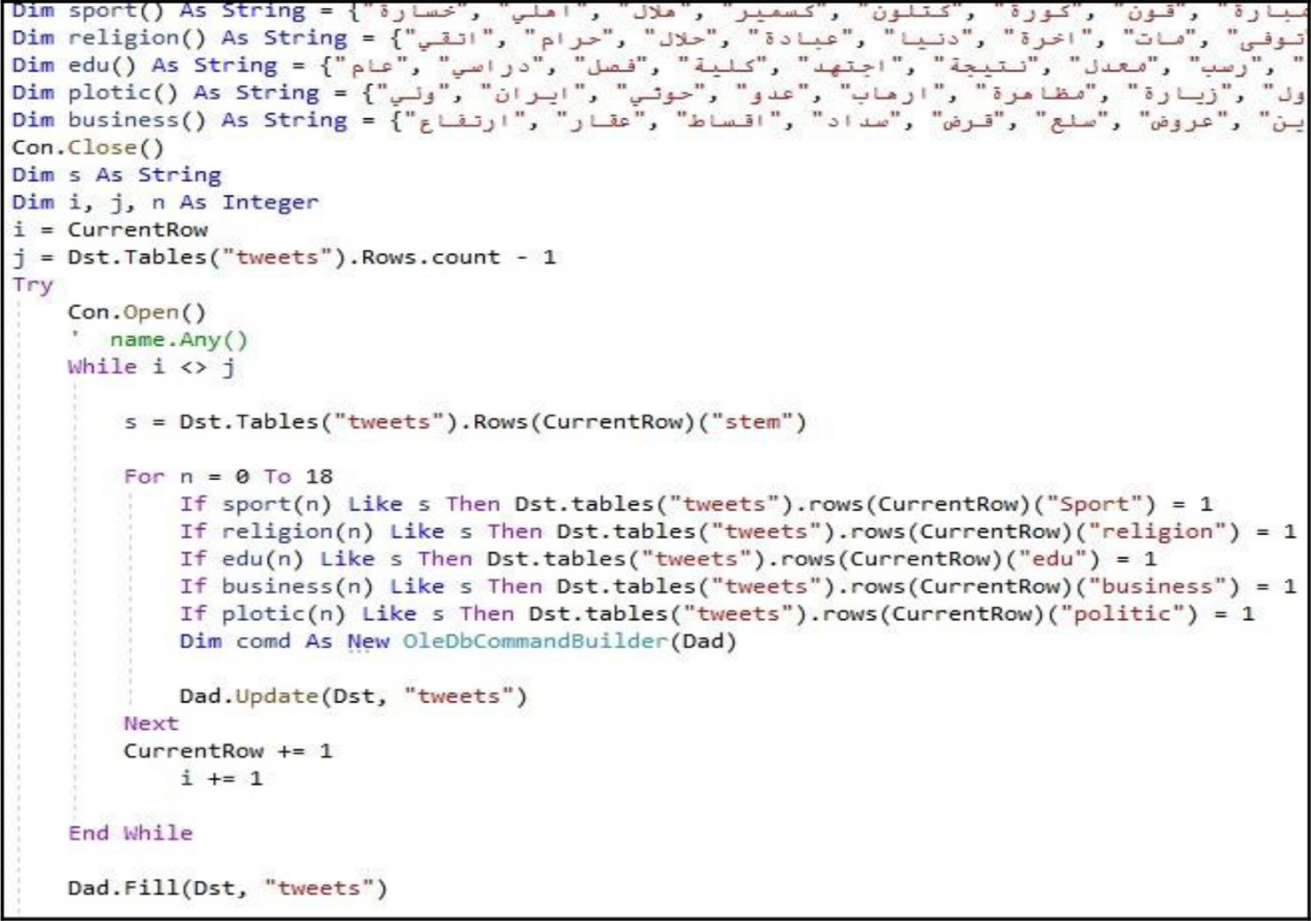
Screen snapshot of the algorithm applied during topic classification processing.

The implementation of our approach results in an integrated application that deals with Arabic text on Twitter. This application collects and preprocesses the data till the processing stage and extracts information from them. Figures 17 and 18 illustrate snapshots of the application that reveal how the integration of these processes is implemented.

The application in this experiment is designed to provide all possible information that can be extracted from the tweets text, starting from simple methods to retrieve information, such as query language, to complex methods such as topic classification and sentiment analysis.

#### 4.5.1 Information retrieval

Availability of the Arabic tweet data in the database table facilitates easy application of any database query and presentation tool for extracting and retrieving required information from these data. As an example, Figure 17 illustrates the selected tweets from Kuwait (location). It can be seen that there are several different ways in which the results can be presented, e.g., visualization methods or reports.

#### 4.5.2 Topic classification

**Figure 19:**
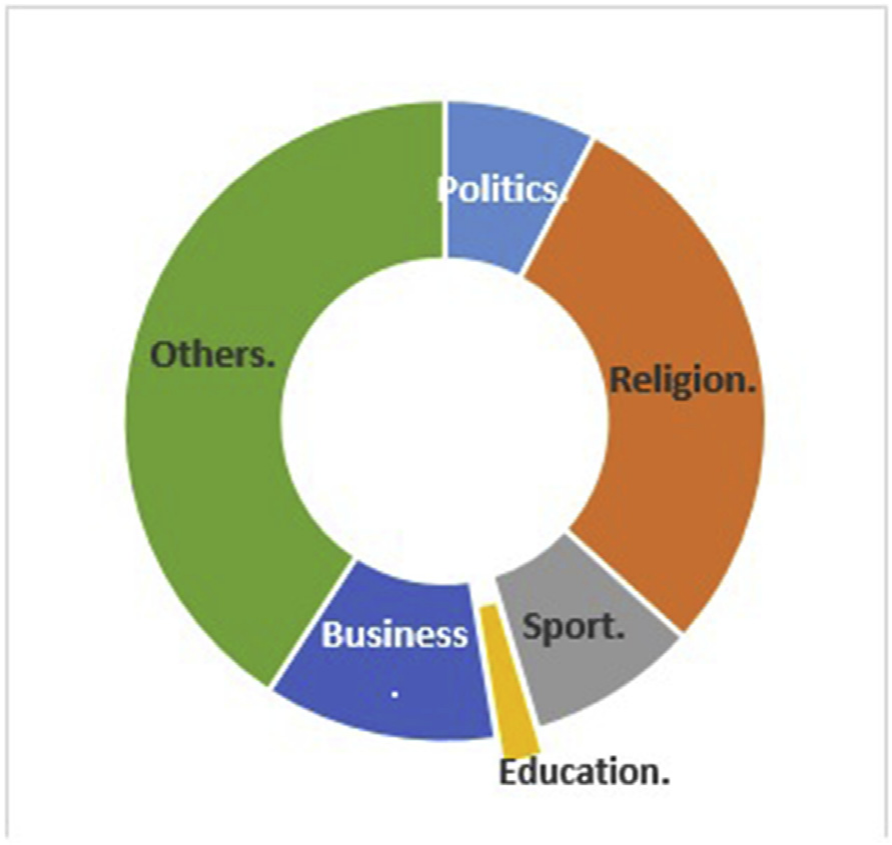
Sample visualization result of twitter topic classification.

Based on our dataset structure, any type of data categorization process can be provided. As an example, topic classification can be provided by using classifier data to identify the topics of the texts. In our experiment, the tweets were classified into six groups: religion, sport, politics, education, business, and others. Figure 19 presents the visualization result of this process.

#### 4.5.3 Sentiment analysis

**Figure 20:**
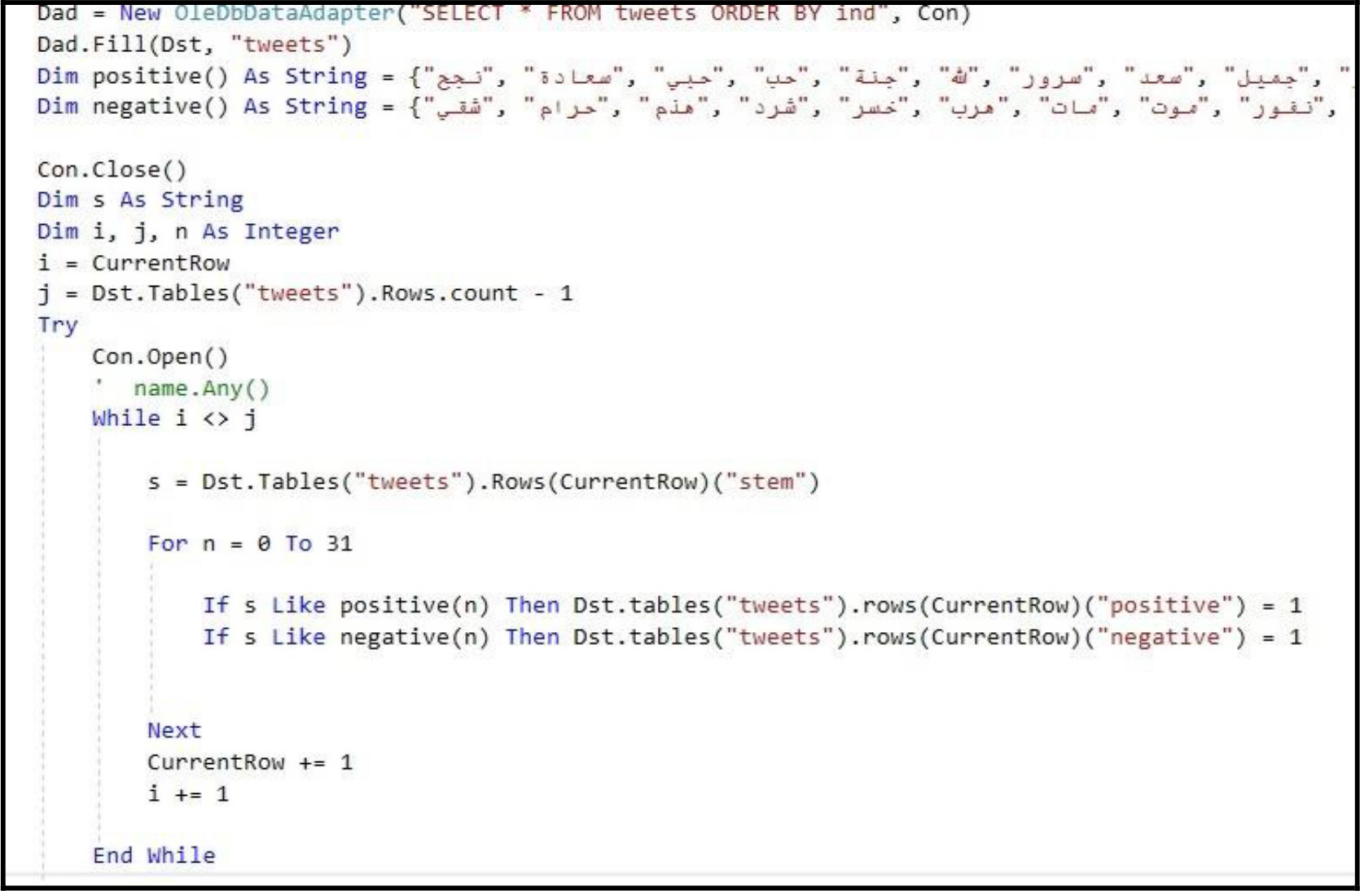
Screen snapshot of algorithm for notating the dataset.

**Figure 21:**
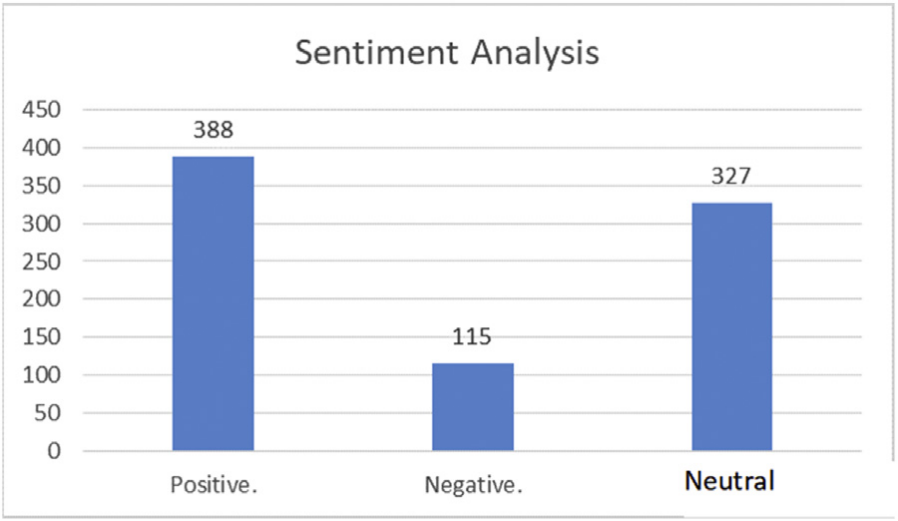
Data notation result.

In our approach, we introduced a new methodology for sentiment analysis. It is based on the database concepts, where the annotation is implemented by searching the text data field for positive or negative words, and then classifying the text according to the majority of positive, negative, or neutral words. The basis of this methodology the text containing positive word/s is positive unless there are negation words; this is also applicable to the negative text. Moreover, the text is annotated as neutral by default. The algorithm for this methodology has two iterations: the first provides the notation without considering negation, and the second checks for negation in the notated text. In case of negation, the algorithm reverses the notation. This method of sentiment data annotation offers the following two advantages over the previous works. The first lies in using the automatic notation, unlike most of the previous works which used manual annotation. The second is in considering the negation, which was either ignored or manipulated by n-gram or other feature manipulation algorithms in previous works. Figure 20presents the screen snapshot of the algorithm used for notating the tweet, and Figure 21 shows the result of this algorithm.

**Figure 22:**
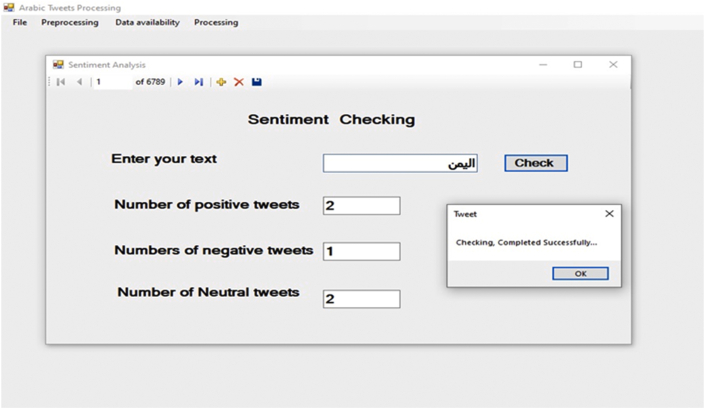
Sentiment checker application and sample result.

This method of dealing with sentiment analysis can help in providing an additional process. In our application, we implemented a method that allows users to know the sentiments of Arab tweeters regarding a subject. For example, Figure 22 presents the sentiment towards Yemen.

## 5 Evaluation

This study proposed an approach that can act as a framework for developing applications that extract information from Arabic text on social media. Accordingly, we believe that the evaluation of this approach is in its ability to implement an experiment for real-world problems and provide useful results. This approach when tested and implemented for the Twitter Arabic text yielded useful information.

Besides providing a framework to handle Arabic text on social media, this approach provides solutions for the challenges in preprocessing and application of NLP for Arabic text on social media. The evaluation and comparison of these solutions is as follows.

### 5.1 Preprocessing (cleaning and normalization)

Most of the previous studies clean and normalize the social media text by removing the noises, i.e., removing some words, samples, and/or characters. Therefore, most of their processing involves dealing with each type of noise. Accordingly, up to six complicated approaches may be required [31]. For example, Al-Twairesh et al. [15] used seven processes to clean and normalize the tweeted Arabic text by removing the URLs, user mentions (@user), and emoticons, and then unifying the different shapes of four Arabic letters dealing with each letter as individual cases. In [3], URLs, user mentions, hashtags, punctuations, emoticons, and symbols were removed. Here, two steps were applied to normalize the words, where each step comprises more than two processes. In the first step, the different shapes of five letters were dealt with; each shape was considered as an individual case. In the second step, they dealt with the non-Arabic characters and the elongated and shortened words—an algorithm was provided for each case. On the contrary, most sentiment analysis research works remove only the stop words (see Table 2). Table 3 presents a summary of the comparison between the preprocessing in our approach and that in the previous studies.

**Table 3:** Comparison of the preprocesses in our approach and those in previous studies.

Study	Approach		Cleaning (Items removed)							Normalization			
			URLs	Other Links	User mentions	Emoticons	Other samples	Non-Arabic letters	Stop words	Character shape	Repeated letters	Non-normal words	Sapling mistakes
[3]	Arabic microblogs' retrieval (Algorithms + dictionary base approach.)		√	√	√	√	√	Some		√	√	character 4-grams	character 4-grams
[15]	Arabic sentiment analysis corpus (dataset development approach)		√		√	√	√			√			
[19]	Social media content analysis (Text mining approach)				√				√				
[20]	Social media user behavior analysis (feature extraction approach)		√	√	√	√	√	√	√	√	√		
[21]	Corpus for Arabic sentiment analysis (dataset development approach)			√		√	√					Removing non-translated words	Removing non-translated words
[22, 23, 24]	Sentiment analysis (ML approach)								√				
[25]	Sentiment analysis (ML approach)		√				√	√					
Our approach	Frame work	The Frame work	Available (users or analyzers can identify the items)							√	√	Some	
		experiment	√	√	√	√	√	√		√	√	Some	

Although most previous studies have invested considerable efforts in cleaning and normalizing social media text, there are still no accurate solutions [34]. We believe that the approaches used in such studies present several difficulties that lead to inaccurate results. These difficulties can be summarized as follows.

(i) It is not easy to detect all noises. Moreover, new noises arise, given the varieties of social media entertainment (variety of cultures, societies, events, and variety in users' age, gender, tendency, etc.)
(ii) Each noise or non-uniformity requires a special algorithm.
(iii) Some noises are difficult to eliminate, e.g., complicated URLs, some types of movements and images, emoticons, signs. Therefore, most of the cleaning processes are performed manually.

In this paper, we presented a new approach for solving these difficulties, as explained in section 3.2 and in the experiment in section 3.3. We proved the correctness of our approach through the cleaned text with uniform letters provided by it. The algorithm designed for the text cleaning has high flexibility, where it can provide clean pure Arabic text or text with additional components because the only change between both these text types is the array that is used for selecting the required items (Figure 9). Furthermore, we used arrays and unified database field types to avoid incorrect results owing to unicode transformation format (UTF) or the right-to-left.

Regarding the normalization in our approach, we provide most of the normalization during the cleaning process, where all the problems of uniformity of Arabic characters are solved in the cleaning phase. The remaining normalization considers repeated characters (elongated letters), extra spaces, and some common incorrect words (words with additional letters or words with spelling mistakes).

### 5.2 Text processing (tokenization, stemming, and morphology)

The difficulties in Arabic word processing in social media text have caused several researchers to use dictionary-based or machine learning algorithms rather than NLP processing algorithms [3, 8, 35].

In our approach, we tried to address these difficulties without relying on dictionaries or machine learning algorithms. To this end, we used two stages after the cleaning and normalization stages. They are the tokenization and the stemmer and root generation phases.

In the tokenization phase, we consider the first part of the problem, i.e., how to obtain the words of the text (section 3.2.2). The results of this stage are two types of words: (i) standard Arabic words free from defects and (ii) non-standard Arabic words, i.e., the words that contain adherent or excessive letters (word elongation), pronoun letters attached to the words, words with spelling mistakes, dialect words, or words of foreign languages written in the Arabic alphabet.

In the second stage, i.e., the stemming phase, we design a special stemmer algorithm to address the abovementioned difficulties and find the stemming and the root of the word (section 3.2.2). This algorithm tests all the words resulting from the tokenization stage. The stemmer algorithm identified each word, whether standard or not (as explained in section 3.2.2).

These processes provided satisfactory results and solutions to the difficulties and complexity of the words of the Arabic text in social media, which is evident from the experiment in this study.

Table 4 presents a summary of the comparison study with the previous works. We can conclude that our approaches provide certain advantages over the previous studies as follows.

**Table 4:** Comparisons between ANLP in our approach and that in the previous studies.

Study	Stemming	Root	Tokenization	Morphology
[3]	Statistical stemming	Not used	Applying previous tools (AMIRA tools)	Dealing with common forms (without using any morphology generation algorithm)
[15]	Not used	Not used	Applying previous tools (Madamira tools)	Not used
[19]	Applying existing stemmer algorithm	Not used. Only recognizes the root inside the word stem (no individual consideration)	Implemented and clubbed with (WEKA) data analysis tool	Not used
[20]	Not used for the dataset only for the topic	Not used	Implemented for all the words inside the topic	Not used
[21]	Applying existing tools	Implemented using existing tools	Applying (POS) tools to categorize words	Applying (POS) tools to categorize words
[22, 23, 24]	Applying existing tools	Not used	Implemented	Not used
Our approach	New approach for social media Arabic text	New approach for social media Arabic text	Implemented	Designing and applying morphology algorithm that generates Arabic word morphology template

Most of the previous studies used the idea of dictionaries (as aforementioned).

Most NLP processing methods such as morphology analysis and stemmer and root generating algorithms are applicable to the CA or MSA text [4, 32, 36].

Although good stemming algorithms such as Khoja’s stemmer, Buckwalter’s morphological analyzer, and tri-literal root extraction [32] are available, most of them are appropriate for MSA, and may not be applicable to social media Arabic text.

In [35], the authors provided a system for subjective and sentiment analysis (SSA) that deals with the tokenization and the complexity of morphology characteristics of the Arabic language lexemes, lemmas, and POS tags. Although, their study is promising, their approach is a machine learning-based one, which is oriented to the subjectivity and sentiment analysis processing without sufficient focus on the difficulties in handling social media Arabic text. Therefore, we cannot compare it with our work.

Moreover, our experimental results proved that applying NLP tools such as tokenization, stemming, root, and morphology generation for social media Arabic text enriches the text and obtains more information (see section 4.3).

## 6 Conclusions

This paper proposes an approach that acts as a framework for producing an integrated application to process and analyze Arabic text on social media. The novel approach mitigated the difficulties in preprocessing and NLP of the Arabic text on social media and extracted valuable information from Arabic social media text.

The experiment in this study revealed that the proposed approach is successful in addressing the difficulties associated with preprocessing and NLP in Arabic text on social media and provides a structured, well-collected, and organized Arabic dataset that was helpful in extracting useful information from the Arabic text on Twitter. Additionally, it contributed in mitigating the challenges in data collection and data preparation in stoical media (see [37]), which is considered as a new addition in the area of big data processing.

The approach provides new methodologies and algorithms for cleaning, normalization, tokenization, stemming, root, and morphology generation. These algorithms can act as toolkits and can be reused or embedded in future work. The dataset generated in the experiment was tested and implemented in several ways, and it successfully provides useful information. Moreover, based on this approach, the experiment presented new methods and algorithms for information extraction, topic classification, and sentiment analysis.

The work presented in this paper throws light on the additional and integrated aspects of preprocessing Arabic text on social media, compared with previous studies.

Although Arabic is a structured language with an abundance of vocabulary, where morphology plays an important role, this issue did not find sufficient focus in most of the previous studies. This study employed several NLP methods to enhance the dataset by providing additional information such as word morphologies and roots that are not presented in the previous papers.

Our approach provides a viable solution to most of the challenges in handling Arabic text on social media, such as subject analysis, sentiment analysis, studies of dialect language, and/or information retrieval.

## Declarations

### Author contribution statement

Mohamed O. Hegazi: Conceived and designed the experiments; Analyzed and interpreted the data; Wrote the paper.

Yasser Al-Dossari, Abdullah Al-Yahy, Abdulaziz Al-Sumari and Anwer Hilal: Performed the experiments; Analyzed and interpreted the data; Contributed reagents, materials, analysis tools or data.

### Funding statement

This project was supported by the deanship of scientific research at Prince Sattam bin Abdulaziz University under research project number 2017/01/7773.

### Data availability statement

Data associated with this study has been deposited at https://figshare.com/articles/dataset/Data_set/12732494.

### Declaration of interests statement

The authors declare no conflict of interest.

### Additional information

No additional information is available for this paper.
